# Clinique Chirurgicale de l'Hôpital de la Pitié

**Published:** 1843-01-01

**Authors:** 


### CLINIQUE CHIRURGICALE DE L'HÔPITAL DE LA PITIÉ. Par *J. Lisfranc*. Tome premier. Paris, 1841, pp. 694.

### The Clinical Surgery of the Hospital of La Pitié. By *J. Lisfranc.*

WE feel great pleasure in introducing to the notice of our readers another practical work, proceeding from the French medical press. It is one of an important character, being the first of a series of volumes descriptive of the practice of the greatest of living French surgeons. It is true that various portions of M. Lisfranc's doctrines and practice have been from time to time published by himself or his pupils in the French medical journals, with no niggardly hand, and have been transferred thence to our own pages; but we have them here in a more conveniently accessible form, encreased by many additions, derived from the author's extensive practice, fully authenticated by his own publication of them, and benefited by his revision. It is to be lamented that every hospital physician and surgeon does not feel it incumbent upon him to present the world with a detail of the practical results his career has enabled him to arrive at; and, indeed, one can hardly acquit one enjoying the vast opportunities that hospitals afford, of neglect of duty, who omits calling, through the medium of the press, his brethren to the participation of the benefits thence derivable.

The size of the volume before us, our limited space, and the importance of presenting to our readers as distinctly as possible the opinions of so great an authority as M. Lisfranc, will prevent our indulging in any observations in the course of this review—confining ourselves to the more laborious, but also more useful, task of presenting to the reader an accurate abstract of the author's views, delivered as nearly as possible in his own words.

No system is observed in the arrangement of the various subjects treated of, and we cannot do better than follow the order in which we find them set down.

#### I. REMARKS ON THE UVULA.

The uvula may offer a great variety of appearances, without any corresponding change of the voice. Thus the author has seen it as a short thick tubercle, or entirely absent, while, in other cases, it has been half as long again as its normal length. It may also be bifid, with or without the same condition of the palatine arch. When it is very thin, or a mere tubercle, dissection proves it to consist only of mucous membrane, and a great number of follicles, while its bifid condition, and the absence of the levator uvulæ, constantly produce a procidence, for the relief of which all medical applications are useless.

Besides the levator uvulæ, covered with mucous membrane, the uvula consists of cellular tissue, thickly studded with small glands, resembling in texture those of the tonsils. Its free extremity does not consist of muscular fibres, but three mucous follicles, susceptible of very great development, may be often found there. The observation of many cases, in which the uvula has been removed, prove that it is not concerned in the formation of the voice, or in the articulation of sound. Lisfranc believes, with Richerand, that it indicates to the pharynx the arrival of the alimentary bolus, the passage of which it facilitates by the quantity of mucus it supplies. Another function may also be assigned to this organ. When we desire to clear into the pharynx the secretions of the nasal cavities, we close the mouth, and introduce into them as large a quantity of air as possible. At the same time the velum palati is borne upwards and forwards, and the uvula, following this movement, and being situated, like the larynx, upon the median line, it forms a protecting barrier to this important part, forcing to the right or left, bodies which would otherwise have fallen into the glottis. He mentions three proofs of the truth of the above statement. First, any one by making forcible inspirations before a mirror may observe the movement described: secondly, that the rudimentary condition of the uvula is most remarkable in animals whose heads incline to the ground, and least so in those whose organization approaches the human, as in the red ourang-outang, which possesses an almost perfect uvula: thirdly, when the uvula is in a condition of complete procidence, or when it has been entirely removed, unless inspiration be conducted slowly and carefully, the nasal mucus passes into the glottis.

*Procidence of the Uvula.*—The influence of the procidence of the uvula in exciting other affections is too often over-looked. It may produce, maintain, or aggravate inflammatory affections of the throat, of the lungs, and of the larynx; so, also, its excision is sometimes the only mode of combating certain cases of chronic gastritis. When the procidence arises from acute inflammation in its early stage, or from chronic inflammatory action, local bleeding, pediluvia, astringent gargles, or the application of nitrate of silver, are the means to be adopted. But when active inflammation, occurring in a robust person, is present, a general bleeding should precede these. When the inflammation has become truly chronic, or the organ engorged with serum, or paralysed, small applications of pepper, ginger, or caustic may be had recourse to. When the affection resists all means, and especially if it puts on a scirrhous character, the removal of the entire organ can alone suffice, for if a part be merely removed the malady is frequently reproduced. The author considers a very needless multiplication of scissors for this operation has been made. Their points should be blunt and their blades curved. Although, in some cases, when paralysis of the part exists, the patient does not suffer pain during the operation, in other cases he does so most grievously. After the removal of the uvula we must carefully treat the persisting chronic inflammation of the throat, or a continued hoarseness of the voice may remain.

### II. ON THE APPLICATION OF LEECHES TO VARIOUS PARTS.

The thickness of the epidermis of the palms of the hands and soles of the feet usually prevents leeches biting in these situations.

The cicatrices often continue very visible, and therefore we must abstain as far as possible from placing the leeches upon parts habitually exposed: and, when this is unavoidable, we must employ very small ones.

In women and children, in whom the skin is very delicate, we must avoid the course of large veins, otherwise hæmorrhage, difficult to arrest, may be produced.

Leeches applied to the face frequently produce œdema and erysipelas.

Applied at the angle of the eyelids they will frequently produce œdematous erysipelas, an ecchymosed condition of the eyelid, and an aggravation of the acute ophthalmia, They should be placed on the temple, along the roots of the hair, and, if necessary, behind the ears, where they will be just as useful. In meningeal affections their application to the mastoid processes is attended with great advantage.

The practice of placing leeches upon the inner surface of the eyelid is bad: in acute inflammation the site is too limited to admit of sufficient blood being abstracted hence, and, even in chronic affections, Lisfranc has often seen the practice give rise to serious aggravation of the inflammatory action.

When leeches are applied to the neck for inflammatory affections of the throat, they give rise to unseemly scars in women. They are equally useful applied upon or behind the mastoid processes, or along the roots of the hair. Moreover, in women, whose skins are very delicate, or in young children, the leech may open one of the superficial veins of the neck, and thus give rise to phlebitis—the more dangerous from the vicinity of these vessels to the heart—or, in other cases, a severe hæmorrhage may call for the use of caustic. The advantage of easy compression behind the ears is obvious.

When leeches are applied to the epigastrium some of them are often placed over the cartilages of the ribs; but the mobility of these parts during respiration often causes a large loss of blood, especially in young children. Lisfranc has seen a life lost through the commission of this error.

When leeches are applied to parts possessed of much *embonpoint,* little blood will be procured. Thus, if you attack a peritonitis in a very stout person with forty leeches, you will but aggravate the disease, by adding to the congested state of the peritoneum. You must double the number, and very often precede their application by a general bleeding.

Avoid the situation of the chief subcutaneous nerves, as the bites will cause excessive pain. Thus, *e. g.* in the fore-arm you must prefer the dorsal to the palmar aspect.

When leeches are applied to the mucous membrane of the vulva, their bites are very painful, and difficult to heal—while, when applied around the region, the same inconveniences do not arise.

Do not place them too near the rectum, for the bites, bathed in matters which proceed from its cavity, degenerate easily into obstinate ulcers.

The application of leeches to the scrotum, prepuce, or skin of the penis is attended with excessive suffering, and has been occasionally followed by gangrene. They should be applied along the course of the spermatic cord, where they succeed equally well.

As numerous nervous filaments are met with on the dorsal aspect of the hand, and on the instep, leeches should not be placed there—but rather upon the posterior and inferior portion of the fore-arm, and the superior and external portion of the leg—the practice of La Pitié shewing that they are just as useful in these situations.

Do not apply leeches to the inferior parts of the leg, for, even when the part is sound, and much more so when there are varicose veins, the bites may produce very obstinate ulcers.

When leeches are placed *upon* the breasts, they cause great pain, disagreeable scars, and frequently erysipelas—while they are equally useful when applied *around* these organs.

Although it is generally admitted that leeches should be applied upon inflamed parts, yet, in erysipelas complicated with phlyctenæ, and in phlegmon, presenting a deep red colour at its centre, if this rule be followed, gangrene will often follow—to say nothing of the severe pain, arising from the exalted sensibility of the parts. Place the leeches around the margin of the inflamed zone.

Gangrene may also be excited by applying leeches to œdematous or ecchymosed parts, and to colourless swellings, in which the skin is indurated or adherent.

Do not apply them to a syphilitic bubo, but at a distance of three or four inches from its base—otherwise, you will sometimes find the little wounds converted into venereal sores.

Leeches must not be placed upon a fractured limb, as the subsequent pressure of the splints may retard the cicatrization, and induce inflammation or ecchymosis.

When you are in doubt concerning the scirrhous nature of a tumor, do not apply leeches *upon* it—for you may find the bites converted into cancerous sores, and the progress of the affection untowardly hastened.

The difficulty there exists in establishing the diagnosis of the *engorgements* of the neck of the uterus, should lead us to proscribe their application to that part.

### III. General Observations on Bleeding.

Experience has shewn that *general* is more efficacious in inflammatory affections of the parenchymatous textures—these being more especially under the influence of the larger vessels; while *local* bleeding is best adapted for inflammation of the membranous tissues—influenced as these are by the capillary circulation.

The loss of blood is supported with less ease in the state of health, than when inflammatory action is present; and so much is this the case that, as the patient approaches convalescence, or the affection becomes chronic, he is found to be less and less able to bear depletion. Inhabitants of warm climates support the loss less easily than those of temperate ones. Idiosyncracies are sometimes very remarkable as regards blood-letting: sometimes, in apparently feeble persons, abundant losses of blood are tolerated with great facility, while even a few leeches will, in some apparently robust persons, at other times produce complete anœmia. Again, in other persons of an ordinary appearance, bleeding to a frightful extent is followed by no ill effects. Women possess extraordinary reparative powers under such circumstances.

When an acute supervenes upon a chronic inflammation, and former evacuations have depressed the pulse, and weakened the patient, we must not bleed again, though the disease still continue, lest we diminish too far the resources of Nature. In the case of old persons we must not fear abstracting blood, due attention being had to their age and constitution, when we find a well-developed inflammation, seated in healthy organs, and manifesting sthenic symptoms : but, when the attack is grafted upon a chronic inflammation, affects a diseased organ, or is attended by asthenic symptoms, bleeding will be fatal, or at least dangerous. In traumatic inflammations, especially if these do not implicate the abdominal viscera, bleeding must be insisted upon; if prompt, the inflammation will soon yield, and the subsequent suppuration and other ill-effects be prevented, or much lessened—for injuries are found to resist the effects of bleeding with less pertinacity, in proportion as it is early applied to them. Although gangrenous inflammation forbids all venesection, yet, supposing that in a robust individual the death of the tissues concerned has arisen from an excess of inflammation, which still continues, and menaces other parts not yet attacked, the gangrene may be limited in extent, and the inflammation diminished in intensity by the use of leeches.

It is in inflammations of the *lungs* and *pleura*, that extensive depletion is best borne, at least, providing no considerable extent of pulmonary tubercle exists. What are we to think of those statistical physicians, who, reasoning from an insufficient number of facts, tell us that these affections should be cured without the abstraction of blood? In repeating venesection for affections of the *head*, we must be careful lest we destroy the equilibrium of the nervous and vascular systems—the former being susceptible of excessive excitement when bleeding is carried too far. Inflammatory affections of the *abdomen* are usually speedily attended with great prostration of strength—arising, the author conjectures, from the effects of the mephitic gases produced by a suspended digestion. These pass from the canal by imbibition, and excite a poisonous effect upon the system, one result of which is observed in the fetid and black condition of the stools occurring in these cases. In such cases, depletion requires some caution.

When *revulsion* or *derivation* is intended to be produced by bleeding, it must not be too abundant. From four to six leeches, but especially a venesection from three to six ounces will suffice. M. Lisfranc lays great stress upon the benefit of derivative bleeding, and blames those surgeons, who, only removing blood when febrile action or a full pulse exist, neglect the fact, that while the loss of three ounces of blood will produce no debility, it yet may be attended with the happiest effects.

When it is desired to produce the menstrual flux, leeches are applied around the vulva, or upon the thighs. When, on the contrary, menorrhagia, active or passive, exists, although the woman be very feeble, it is found that one or more derivative bleedings will relieve her—provided, at least, that the womb be not organically diseased. A few leeches will often create an erysipelas, when a greater number will not do so—so, also, a few will exasperate an intense inflammation, which many would relieve. For the purposes of derivation, M. Lisfranc prefers leeches to cupping.

The author has treated, by small revulsive bleedings from the arm, chronic lesions of the uterus, attended with pain, forcing, and an inconvenient sense of heat. These bleedings have generally caused pain of the head, difficulty of respiration, palpitations, &c., but, the affections of the womb, for which they were instituted, have almost always been relieved. In women, who are habitually liable to a congested state of the uterus, two or three small bleedings, practised during the intervals of the menses, are often attended with great advantage. In some very rare cases, however, the uterus becomes more congested, and the symptoms aggravated, but such cases are very exceptional, and are usually very difficult of cure by any means. White-swellings, when not in a state of acute inflammation, have frequently been much benefited by the increased absorption the application of a few leeches around the base has induced. As derivative bleeding causes congestion of the parts in the vicinity of which it is performed, it must not be practised at the arm, chest, mastoid process, &c. when thoracic or cerebral congestions are to be feared. This mode of bleeding has fallen into discredit with some, owing to the erroneous manner in which they have practised it—namely, by abstracting large quantities of blood, and thus producing an injurious effect upon the nervous system.

### IV. The Application of the Stethoscope in Surgery.

*In Fractures.*—M. Lisfranc considers this instrument of the highest utility in the diagnosis of obscure fractures; and that, with its aid, there can scarcely ever remain the slightest doubt whether a bone be broken or not. The swelling, so often present, does not conceal the crepitus from the stethoscope, and the slightest movement indicates the exact site of the fracture—so that, not only are patients assured against the ill-consequences of a faulty diagnosis, but they are spared the painful proceedings so often otherwise necessary to form one at all.

" *General Rules for the Application of the Stethoscope.*

" 1. When the stethoscope is applied to the fractured part itself, it is almost a matter of indifference whether the plug be retained or not; but when we apply it at a point distant from the seat of fracture, the crepitus will become more distinct if the plug be previously removed.

" 2. The more superficial the bones concerned, the louder the crepitus, which will then be produced by almost imperceptible movements—and, as it is always most distinct just over the fractured part, the exact site of this latter is ascertained.

" 3. Although the crepitus is heard with less distinctness in proportion to the distance, yet, only those who have tried the experiment, can imagine at what inconceivable distances it may be sometimes perceived.

"4. When there is a riding of the extremities of the bones the crepitus is much less distinct; but, if an experienced ear cannot distinguish it with sufficient distinctness, it may easily be rendered more apparent after slight extension and counter-extension have been practised.

"5. The fragments of the compact bones furnish sharp, grating, crackling sounds, which, heard through the stethoscope, distress the ear by their loudness.

"6. The crepitus, resulting from the fractured spongy bones, produces a duller sound: it resembles the action of a file upon pumice-stone, but is interrupted at intervals by louder sounds, somewhat resembling those proceeding from fractures of the compact bones.

"7. Oblique produce a stronger crepitus than transverse fractures.

"8. If fluids be effused around the fragments, the crepitus is accompanied by a noise resembling that produced by the foot in a bad shoe that has let in water.

"9. When the fracture is attended with splinters, besides the ordinary crepitus, there may be heard a sort of crackling, as if produced by several hard, angular bodies rubbing against each other.

"10. When there is also a wound of the soft parts, the crepitus is accompanied by sounds resembling those produced by making forcible expirations and inspirations, the mouth being kept wide open the while.

"11. Luxations cannot be confounded with fractures, for the noise produced by the displaced articular surfaces is slight, very limited in extent, and of a duller character—that, in fact, of two moistened and polished surfaces playing upon each other.

"12. The sliding of the tendons in their sheaths produce full, dull, interrupted and infrequent sounds, very unlike those resulting from a fractured bone." 54-56.

*Special Rules* for the detection of each fracture are furnished by the author, but they are too long for our purpose. We will only observe, that they serve to shew how immensely the diagnosis of fractures in the neighbourhood of joints, &c. has gained by the stethoscope, and that M. Lisfranc impresses upon his readers the great importance of educating the ear by frequent experiments upon the dead.

*In Urinary Calculi.*—"To obtain the most distinct sensations, we place the stethoscope, having removed the plug, upon the body of the pubis, or upon the posterior part of the sacrum. If the sound be introduced into the bladder, void of urine, and containing no calculus, the motions imparted to the instrument produce a noise like that of a pump in action. When there is a little urine in the bladder, a sound like that made by working the saliva in the mouth, is sometimes produced: but, if a stone exists, a very distinct tinkling is heard, or else sounds resembling those resulting from filing a compact or a porous body. When, as an experiment, some soft tissues have been introduced into the bladder, sounds similar to those heard in the empty bladder, or when it contains only a little urine, were perceived." 77.

*Biliary Calculi and other Affections.*—M. Lisfranc has long thought that the stethoscope might be useful in detecting biliary calculi, and has tried many fruitless experiments in these cases. In one alone could he detect a crepitus. He considers the instrument of utility in detecting foreign bodies, when sonorous, lodged in the ear, nares, pharynx, œsophagus, rectum, vagina, womb, sinuses, the track of a wound, &c. &c.—as also in ascertaining the existence of sequestra in necrosis.

### V. Observations on Fractures and their Treatment.

Excepting when the attendant inflammation is excessively severe, surgeons are accustomed at once to apply the apparatus to fractured limbs—a practice of which M. Lisfranc disapproves, not because he agrees with Troja that this early compression retards the deposition of the provisionary callus, but because it may easily produce sphacelus. In the very simplest cases of fracture he applies the splints at once, but removes them, for examination, on the next day at very latest—always bearing in mind the ease with which gangrene may be produced in some subjects, and this not only in the aged, but also in some young persons of apparently robust health.

When however the fracture is of a rather more complicated nature, no apparatus is to be applied until the fourth, fifth, or even the sixth day—and the author appeals to the success he has met with at La Pitié during fifteen years, as a proof that this is sound practice. If, however, the patient becomes delirious, and it is found impossible to manage the limb by strips of bandages, the splints must be had recourse to, upon the principle of choosing the least of two evils. But in cases of bad fracture, besides this late application of the apparatus, he adopts other means.

" I order a bleeding from the arm, to a greater or less extent, according to the constitution of the patient; and the limb is covered with an emollient cataplasm, renewed four or five times a day. The most rigid diet is enjoined, and the extremity is maintained in a convenient position by several napkins folded lengthwise. If the pulse is good, the countenance not pale, and the system undepressed, a second bleeding of rather less extent is practised eight or ten hours after the first. Next day, if the state of the circulation permits, I again bleed, though to a still less extent; and, in fact, for three, four, or five days, when no wound exists, and the patient is not too much enfeebled, I continue to draw small quantities—from three to six ounces. I choose in these latter bleedings a point as far removed as possible from the seat of inflammation—adding, thus, to the benefits of depletion those of revulsion. As already observed, in traumatic inflammation, depletion is far better borne than in other forms. Venesection is preferable to leeches. We have to combat inflammatory action, and a more or less considerable effusion of blood. According to the experiments of Magendie, not only will the general bleeding relieve the phlegmasia, but, also, by suddenly diminishing the quantity of blood contained in the veins, it will render them more eager for liquids, and the absorption of effused fluid will become more rapid, than it would be if you applied leeches, which act only upon the capillary system. If you do not admit the correctness of this reasoning, at all events do not apply them to the fractured limb itself—for we have often observed, in patients brought into the hospital, the bites, subjected to the pressure of the splints, converted into ulcerations, which, increasing in size, have frequently offered much obstruction to the subsequent use of the apparatus necessary for the consolidation of the fracture. When, however, the fracture is complicated with wounds of the soft parts, you must be on your guard—for it is essentially necessary to discontinue the evacuation of blood when the secretion of pus is about to commence; and the neglect of this fundamental therapeutical principle has cost many patients their lives. Can it be necessary to state, that if you abstract blood, during the secretion of pus from a recent solution of continuity, you risk much the production of a resorption of the latter fluid—the frequently fatal results of which are well known. Even a very low diet must not be continued when the secretion of pus has commenced, unless the deranged condition of the alimentary canal prevents the administration of all descriptions of food. Irritation or inflammation of the stomach may be induced by hunger itself; and the patient, if fever be not present, would be living as it were upon his own system, the function of absorption rendered more active, and the resorption of pus more probable." 86.

To the observance of the above precepts the author attributes the great success that has attended his practice, and the fact of his having been able to preserve limbs from amputation in so many cases of bad injuries to joints, &c. It is erroneous to suppose that the delay recommended causes the patient more suffering; nor is the cure retarded, for callus is not deposited during the existence of the effused blood or inflammatory action.

During the first, or even second week, the apparatus must be removed every or every other day, by which means alone are we able to counteract any vicious consolidation. It is to be remembered, that, at first, the muscles, much irritated by the violence to which they have been subjected, resist all our adjusting processes, and it is not until such irritation has diminished that you can attain your ends. And, although the callus becomes more and more solid from day to day, it will yet yield to means perseveringly adopted to prevent deformity—so that we must not think a displacement, against which we have struggled in vain for several days, as necessarily invincible.

As to the removal of *splintered portions of bone;* when they are not very large, and maintain adhesions with the soft parts, we should not remove them—for Ambrose Paré has shewn, and subsequent experience has frequently proved, that they may continue to live, and become rejoined to the bone whence they had been entirely separated. In the case of an old woman, who died from other causes, a large portion of the tibia, two and a half inches long, which had become detached, was found living, and completely re-united to the remainder of the bone. When the splinters are detached from the soft parts, we may endeavour to extract them, to effect which we may have to make some incisions; but if these are likely to be required either very deep or very long, we must renounce the attempt, as risking serious accidents. When these splinters have been left to themselves, they are sometimes found, in subsequent autopsies, to have become encysted, and at other times to be more or less surrounded by a deformed callus; in some cases they penetrate among the soft parts to great distances from the seat of fracture, and in others they become absorbed. The author is acquainted with a great number of the old officers of the army, who suffer from time to time considerable inconveniences from the irritation and inflammation these splinters keep up. He prefers treating such cases in a temporizing manner by rest and antiphlogistics, to employing the large incisions sometimes recommended.

When the splints have been removed, and the fracture become consolidated, you must still see your patient frequently, at least for the first two weeks after—otherwise you may find a serious deformity afterwards appearing—a deformity, however, in the great majority of cases, susceptible of relief, although it may have existed several weeks.

The effusion of blood sometimes resists general bleeding, ordinarily so efficacious in removing it. In such cases Lisfranc has employed *diuretics*, upon the same principle he would use them in dropsy, with the best effect. So, too, purgatives are here highly useful, when the strength of the patient, and the condition of the alimentary canal admit of their exhibition.

*The Starch Bandage.*—M. Lisfranc objects to the immediate application of this in simple fracture without displacement—as, after the subsidence of any tumefaction or infiltration, the portions of the bones may become displaced, and yet we are unable to ascertain that this is the case. To ensure the case doing well, he considers the daily inspection of the part is necessary, which may be obtained by making a longitudinal section through the bandage—and with this precaution he adopts the starch apparatus in simple cases. Where, however, there is displacement of the ends of the fractured bones, the swelling which is present may prevent the surgeon assuring himself of the exact adaptation of the parts ; and, after such swelling has subsided, or, by reason of the atrophy resulting from long-continued pressure, a considerable interval may be left between the apparatus and the limb. The exact contact of the parts may also disappear during the application or drying of the bandage. But if the fracture be very oblique, even by aid of an opening in the bandage, how are we to re-adjust the parts when displaced, or how apply any additional compresses or splints that may become necessary ?

The number of badly united fractures after the use of the starch bandage that the author has met with confirms his objections to it. It should not be employed in any case having a tendency to displacement, until the callus has become sufficiently solid and straight to prevent any fear of a vicious direction resulting. It is also objectionable when any wound of the soft parts complicates the fracture—especially from the possibility of the occurrence of suppuration, and the difficulty of giving issue to the pus. It frequently occasions by its hardness irritation and excoriations of the skin—a circumstance of some consequence in the aged. Patients commit a great error by attempting to use their limbs too soon after the application of this bandage.

The author alludes to two cases of *Fracture of the Spine* in which the advantage of the antiphlogistic treatment was conspicuous. Each patient was submitted to nineteen bleedings, and the application of many leeches within twenty-one days—accompanied by a rigid diet. In one case the paraplegia was removed, and in the other, the patient could walk upon crutches.

In *Fracture of the Ribs* the bandage, as ordinarily applied, by acting exclusively upon the transverse diameter of the thorax, often assists in thrusting the broken ends of the bones inwards. M. Lisfranc applies compresses of two or three inches in thickness along the whole length of the sternum. Compression now acts from before backwards and *vice versâ* ; the arc of the thorax is augmented, and the fractured bones are carried outwards.

In fracture of the *Surgical Neck of the Humerus*, the pad must be introduced to the very summit of the arm-pit, for, if it is merely placed against the anterior and posterior borders of this cavity, its pressure will prove much more painful, and yet will not act upon the superior portion of the fracture at all, and upon the inferior but slightly. In regard to the *position* of a fractured limb, Lisfranc considers that, with few exceptions, the half-bent is the best. One of the exceptions is the case of a fracture situated at the upper third of the leg, near the condyles of the tibia. If we attempt to place the limb in the semi-flexed position, the upper portion of the fracture is immediately displaced by the action of the semi-tendinosus, semi-membranosus and gracilis muscles. There are cases, also, in which, by reason of the peculiar position of the broken ends of the bone, the extended position may be required, although in almost all similar accidents the semi-flexed is the most advantageous : *e. g.*—in fractures of the lower-third of the leg.

### VI. On Superficial Cancers, usually believed to be deep-seated.

Lisfranc's attention had long been directed to the pathological fact, that cancer does not invade at once all the various tissues of an organ attacked by it. Thus, in the stomach, *e. g.* its ravages are sometimes confined to the mucous, to the cellular, or to the muscular tunic of that organ ; and, even when all the tissues seem equally involved, a careful dissection will usually point out which has become first affected. So, too, in the examination of the bodies of a great number of women who died of cancer of the breast in the Salpêtrière, the pleura, though in contact with the disease, was found unaffected. These, and other facts induced the author to enquire, whether the ravages of cancer in an organ might not be curtailed, especially if its tissue formed part of any peculiar structure, fitted to isolate the malady. If so, the removal of the superficies, performed in time, might save the remainder of the organ. He first put the idea into practice in a case of cancer of the penis.

"I knew," says he, "that of all operations the amputation of the penis is the one the most repugnant to a patient's feelings. It would seem, indeed, as if a man staked all his importance upon the possession of this organ ; the melancholy into which the idea of this mutilation plunges him, proves this to be the case. A very remarkable fact is, that the aged man seems to suffer equal chagrin with one in the prime of life. When these patients survive, which is by no means frequently the case, they never manifest that feeling of gratitude towards the surgeon, so commonly seen in patient's who have been operated upon : they shun him as at once the author and the witness of the degradation to which they believe themselves to have been subjected to." 117.

He began his operation with what he calls an exploratory incision, in order to examine whether the corpus cavernosum yet remained in a healthy condition, so as to admit of the organ being saved. Finding this to be the case, he removed the cancerous part by a "long, painful, and difficult, dissection," and the patient perfectly recovered, performing afterwards his generative functions in a satisfactory manner. In the next case, the testes and cord were laid bare by a laborious dissection, the penis almost detached from the body of the pubis, and the cancer pursued to the roots of the corpus cavernosum, which, " exposed, and as if prepared for an anatomical demonstration," shewed here and there signs of cancerous deposition that had to be removed by the forceps and scissars, the surface being afterwards well scraped with a bistoury. The cure was perfect, and the extent of cicatrization such as can only be imagined by those accustomed to the wonderful reparative powers of nature. Many cases, very formidable in appearance, have been similarly treated by the author. The same success has, also, often attended his operations for cancer of the tongue, the vagina, the lower extremity of the rectum, and the parts surrounding the orbit. Cancer of the *nose* almost always spares the cartilages, and, by careful dissection, the ablation of the organ may be avoided. Moreover, the skin in the vicinity of the solution of continuity produced by this operation, possessing slight mobility, yields but little to the traction of the cicatrix —whence a cicatrization at a very small expence of integument results; and, by taking care to repress exuberant granulations with the nitrate of silver, very little deformity will ensue.

### VII. Some Observations on Cancer.

That cancer is not *contagious*, M. Lisfranc is entirely convinced.

He also denies that there is any *general infection of the system* in this disease. He supposes the disease arises from the development of a greater or less number of tubercles of a malignant nature, which, like ordinary tubercle, may be more especially confined to certain organs. As suppuration attacks and removes tubercles, not simultaneously, but in successive attacks, so cancer might be removed by several successive operations.

From observation upon man and experiment upon animals, M. Lisfranc believes that cancer may be produced solely by common irritating agents. Thus, ulcers, cicatrices, accidental tissues, frequently become cancerous when irritated, while instances of relapse after the removal of such, are very rare. Many persons are very liable to the formation of pimples upon the face, consisting of accidental tissue. No change usually occurs in these until old age approaches, when they either spontaneously ulcerate, or do so in consequence of the irritation of external causes. The pus is scanty in quantity, and, concreting into a thin crust, the patient takes no notice of it. But sooner or later the cancerous condition supervenes if it have not already commenced with the first ulceration. Persons, who are liable to the production of permanent pimples upon the nose, should be careful in not exciting their cancerous degeneration by a too rough use of the pocket-handkerchief. Razor wounds in such subjects often produce dangerous ulcerations. As soon as ulceration occurs, if it be not yet cancerous, apply the nitrate of silver, following its use up by mildly stimulating dressings. Even when the ulcer is cancerous, caustic is sometimes used with good effect; but the author has seen such frightful aggravation result from its employment, that he prefers at once removing the part with the bistoury. If a superficial ulceration of a cancerous aspect be seated, not on accidental tissue, but upon a normal texture, somewhat indurated, it may be touched lightly with the liquid proto-nitrate of mercury—with the intention, however, rather of changing its mode of action, than of effecting the destruction of the tissues upon which it is seated. In this way you will usually effect a cure, especially if you have premised depletion, or afterwards employ it, if any inflammatory action seem to demand it. Fissures of the lips, which, when neglected, so frequently degenerate, rarely do so if they are touched with the nitrate of silver. The same substance is also very useful in the case of a slight erosion of the cheek, covered by a thin, greyish crust. The irritation produced in the faces of old persons, by the accumulation of the matter of the sebaceous follicles, may eventually terminate in cancer.

Whatever book-doctors may think, M. Lisfranc maintains that all experienced surgeons must deny that cancer is always well characterized. In this way he explains Desault's supposed cures of cancer of the rectum, and cites cases of apparently incurable cancer yielding to means seemingly inefficient. He observes, also, that we pronounce many ill-looking sores to be cancerous, merely from the localities whereon we find them, and that we should never so consider them if seen on other parts, *e. g.* the leg. It is well known, also, that syphilis frequently simulates cancer, and the author has often, in doubtful cases, employed anti-syphilitic remedies with the best effect.

The *fragility of the bones* in cancer must be rare, for Bayle did not meet with it in his numerous autopsies. During 18 years M. Lisfranc and his pupils have examined immense numbers of persons dying of cancer, brought from the Salpêtrière, and he has often observed the bones to be anormally soft—so that two or three movements of the saw would suffice to divide a femur.

M. Lisfranc is not deterred from operating, even when cancer has arrived at an *advanced stage*.

"When the viscera are in a healthy condition, the patient not too far enfeebled, and the whole of the disease can be removed without leaving a wound of dangerous extent, sound surgery demands an operation, of which I have had proofs both in Dupuytren's practice and in my own. If art attempts nothing for the unfortunate beings suffering from advanced cancer, a certain death attends them —and what a death! If, on the contrary, an operation be practised, indisputable facts prove that even a permanent, but at all events a temporary, cure may be obtained." 141.

Relapses in ordinary cancer are not frequent enough to allow the opinion of those who denounce operations altogether to have any weight. There is, however, considerable difficulty in establishing in what exact proportion they do occur. The author adds upon this subject:

"I have practised surgery in the hospitals for nearly twenty years, I have paid much attention to cases of cancer, and have operated for it a very great number of times. I have observed the patients afterwards, and have caused them to be observed by others, as far as lay in my power, and I believe that relapse is not a very common occurrence—especially, if, after the operation, means to prevent the reproduction of the disease are adopted by surgeons, who have hitherto too much neglected these. Cancers of the skin, and those developed upon accidental tissues are the least likely to recur. Local bleeding alone will sometimes suffice for the cure of cutaneous cancer; but a persevering trial of this means, in cancer seated in other localities, has not been attended in my hands with any success." 142-3.

Most practitioners admit that cancer is *hereditary*: but, should this contra-indicate an operation? Where the cancerous affection is at once and distinctly developed, its having pre-existed in the family singularly encreases the chance of a recurrence. But the author has observed such chance to be infinitely less, when the cancer has supervened upon chronic inflammation (especially of the cervix uteri), or upon the neglected condition of simple ulcerations. Accordingly as these primary affections are treated, cancer will or will not become developed; and a proof of the truth this statement may be seen in the diminished number of cancers of the womb, that are observed since the publication of modern works upon the diseases of that organ.

It is generally stated that *enlarged lymphatic glands*, placed near the cancer, and that in such a manner as to prevent their removal, present a contra-indication to the performance of the operation. If, however, these glands are not too voluminous, if they are few in number, non-adherent, and their substance not too hard and irregular, M. Lisfranc counsels an operation—referring to the great success he has met with in dispersing these bodies, after the operation, by antiphlogistic treatment. All enlarged glands are not cancerous, or even scirrhous. Those most distant from the tumor exhibit merely an encrease of their normal tissue, while those in its immediate vicinity have undergone the cancerous degenerescence. The tumors, which are sometimes developed under the cicatrix, will often, in like manner, yield to suitable means.

In a case of cancer, with or without ulceration, are we to consider the *whole substance of the tumor* as being occupied by scirrhus? While Lisfranc was pursuing his experiments on the cure of cancer by local bleeding, he observed the tumors so treated underwent a notable diminution at their circumference. This induced him to make careful pathological examinations in some fatal cases; and, although in a few of these, the whole tumor seemed to be completely occupied by the cancer or scirrhus, yet, in the great majority, the morbid change was confined to the centre. The scirrhous nature of the swelling gradually changed into the common inflammatory induration as the circumference was approached. From these facts he deduces some practical proceedings. Thus:—a cancer is of too great an extent to admit of an operation, for the patient would succumb during the healing of the large wound, necessary for its removal. Leeches, proportioned to the degree of strength of the patient, must be applied around the base of the tumor, following them by a large emollient cataplasm, and enjoining a careful diet. As long as the constancy of the pain, and the augmented heat denote the continuance of sub-inflammation, these means must be repeated—the chronic nature of the affection, and the enfeebled state of the patient, however, admonishing us that their repetition must neither be too hasty or too vigorous. In a few days we may have recourse to resolvent applications, and especially compression—yet avoiding the reproduction of inflammatory action. By such means, carefully adopted, and extended over a sufficient space of time, cancerous tumors have been frequently reduced at La Pitié from a size represented by 10, to one of 6 or 4; and, not only has the operation then become practicable, but no more than an ordinary wound has remained after its performance.

Again: a cancer may not be excessively voluminous, but it may involve such important parts as to render its removal difficult or impossible. By the means mentioned the lessening tumor retires from the vicinity of the nerves, vessels, &c. which may have presented the obstacles to the operation.

Some practitioners, observing the partial diminution of the tumor produced by these means, and ignorant of the principle upon which they have been recommended, reject the operation altogether, vainly believing that they can dissipate the cancer altogether in the same manner—thus giving time for the disease to make fatal progress.

After the operation for cancer the fingers must be carefully passed over the whole surface of the wound, by which means small tubercles, easy of removal, may be often felt—they sometimes being completely imbedded in the pectoral muscles. When very numerous they can rarely be completely extirpated, and when left they frequently give rise to the reproduction of the disease. In patients dying a few days after the operation, the author has found hundreds of these bodies, even under the scapula and clavicle, and upon the pleura.

Observation proves that cancer is liable to relapse, in proportion as its progress has been active, or it has been complicated with acute inflammation. This last is to be met by local depletion, continued for some days prior to the operation. Relapses will be less likely to occur, also, if half an inch of sound skin be included in the incision—leaving, however, enough integument, when it is sound, to procure union by the first intention, thereby diminishing the extent of irritation of the wound and subsequent cicatrix. The surgeon's care of his patient must not terminate with the production of cicatrisation, but also be directed to the prevention of a relapse, which, when patients are docile enough to obey the directions given them, may usually be accomplished. They must be told, that, even when the wound has healed, the cure is only in part completed. All causes likely, whether mentally or physically, to interfere with the health must be sought for and removed.

In persons of a sanguine temperament, or when suffering from the obstruction of some flux, let bleeding be practised; and, if the parts in the vicinity of the cicatrix become congested, a revulsive bleeding of three or four ounces must be employed. The vitality of the susceptible organs must be modified by the use of powdered hemlock, (the extract usually being very bad,) for many months. Beginning with a grain every morning, the dose may be gradually augmented to four. Lisfranc has a very high opinion of this remedy, as a solvent and anti-nervine—and its utility is also well seen as the latter, in the gastralgiæ of women suffering from uterine affections. Occasional mild aperients, exutories, in case of retrocession, and, after omitting the hemlock, the external or internal use of iodine, are other useful means. Much good also results from compression, employed by means of agaric and a bandage, and extending beyond the cicatrix. The cicatrix must not be irritated, especially by too early a movement of the parts. The mildest diet, and, if debility be not present, even abstemiousness, should be enjoined.

### VIII. Malignant Pustule.

The author agrees with Enaux and Chaussier in dividing the progress of this affection into *four* periods. 1st. When the disease is seated in the mucous tissue of the skin—characterised by itching, the formation of a vesicle, and the flow of a brownish fluid—and continuing usually from 24 to 48 hours, but at other times for a far shorter period. 2nd. When it affects the chorion. This period lasts for two, three, or four days, but in some cases only for a few hours, and is attended with the formation of a tubercle, and vesicular areola, and encreased itching. 3rd. When it extends to the sub-cutaneous cellular tissue. In robust subjects, four days may be passed in this stage, but in others a much less time. All the symptoms are aggravated, and much swelling of the part occurs. 4th. When it becomes propagated to the economy at large, producing general symptoms—with which, in other cases however, the disease may commence. In some cases the various periods follow each other with such rapidity as to become confounded, and the whole progress of the affection may not then occupy 24 hours.

The septic and debilitating character of the malady calls for tonics and stimuli from the commencement; and we shall do wrong to await the supervention of the general symptoms. Quinine, either alone, or conjoined with polygala or serpentary, forms the best tonic. These medicines are contra-indicated, when there is a dry, red tongue, or tender belly. Ipecacuanha relieves the "embarras gastrique" efficiently, and where the intestines are also at fault, a laxative may be joined. Bleeding must be entirely discountenanced. Lisfranc once saw the case of a robust young man, in which some leeches had been applied prior to the appearance of any general symptoms. The blood poured fast from the bites, and the patient died next day.

M. Lisfranc, observing that the caustic applied to the gangrenous parts and their *immediate* vicinity, by reason of their destruction or debility, did not produce sufficient re-action to raise a "barrier of inflammation" to the progress of the disease—conceived the idea of applying the actual cautery, here and there, around the disease, but at a distance of two, three, or four inches, from its margin. In this way a larger surface would be implicated, a higher degree of salutary irritation produced, while, in parts not yet attacked by the disease, and retaining their vital energy, the requisite re-action would be more likely to occur.

Great success has attended this practice. Where this is objected to, he binds over the centre of the part for a few hours, in an early stage of the affection, a small portion of potassa fusa, or butter of antimony, renewing them if necessary, and dressing with digestive ointment. The action of caustic substances is favoured by the use of the chloride of sodium.

### IX. The Removal of the First Dressings after Operations, &c.

M. Lisfranc, having observed the great sufferings patients endure during the dressing of their wounds, when this is delayed until the third day, arising from the constriction of the bandages, the adhesion of the lint, plaisters, &c., agglutinated by dried blood—to which may be added the frequent production of erysipelas—has been long in the habit of merely applying after the operation a pledget, in which holes have been cut, well spread with cerate, and extending far beyond the edges of the wound. This he renews at the end of twenty-four hours, without pain or inconvenience to the patient. In the present work he is chiefly employed in proving his priority in this practice, since adopted by others without acknowledgment; but he takes occasion to observe, that he continues to have daily opportunities of witnessing its excellent effects.

### X. On Sprains.

These occur especially in the ginglymoid joints. Their ligaments are numerous and strong, and although, by the resistance they offer to external violence, they frequently prevent luxation, yet the traction and torsion to which they are subject render them very liable to become sprained. The parts of the orbicular joints are so differently disposed, that, while their movements are more varied and extensive, their liability to displacement is greater, and the sprains occurring in them are slight and infrequent.

Even a simple sprain, when neglected, may lead to a serious secondary affection, especially in the scrofulous, gouty, rheumatic, or syphilitic subject—in whom it may, as also sometimes in even healthy individuals, terminate in true white-swelling.

Refrigerants are useful when applied very soon after the accident; but they must be applied without intermission, (lest an injurious re-action be set up,) and discontinued as soon as inflammation is observed, which they will only aggravate. The application of bandages, steeped in cold and astringent fluids, and the use of irrigations, are disapproved of by the author. As soon as the practitioner sees the patient, whether cold has been had recourse to or not, and even during its application, he must bleed him from the arm, for the purpose not only of counteracting inflammatory action, but also of facilitating absorption of the effused fluids. Leeches are not proper, and especially when applied to the ecchymosed parts, where they may cause gangrene. If the subject will bear it, the bleeding is repeated two or three times in the twenty-four hours, and afterwards revulsive depletion is to be practised at some distance from the seat of injury. A large emollient cataplasm is the best local application. In five or six days the pain will have disappeared, the effusion become absorbed, and a mere œdematous condition of the parts remain. Discontinuing the above means, we now resort to compression by means of agaric and a circular bandage—properly adjusted, however, according to the peculiarities of the joint affected, or the swelling may become encreased rather than diminished. In this way the most violent sprain may become cured in fifteen or twenty days, and the joint restored to its normal volume and movements. Another week may elapse before gradual attempts at walking are allowed.

None of that debility of the joint, which so often leads to relapse in the common mode of treating the affection, is present. In some cases, a greater degree of pressure, by means of graduated compresses, is required. In others the effusion resists all these means, when diuretics may be prescribed with great success; and, in feeble subjects, these medicines may be advantageously substituted for the bleedings. Purgatives are also of great utility.

### XI. On Burns.

Life may become endangered after burns during the period of irritation, that of inflammation, that of suppuration and the resorption of pus, and from prostration. The author recognizes five degrees of burns, according to the extent of injury inflicted. He has found the solution of the *chloride of soda* an excellent application. It acts as an astringent and sedative, affording rapid relief to suffering, preventing an encrease of inflammatory action, and dissipating this, when it already exists. When there is a solution of continuity, without an eschar, it possesses in a great degree the power of inducing a plastic exudation, which becomes at once organized, as it were, like a false membrane. It is developed from the circumference to the centre, and rapidly closes up the solution of continuity. It will produce the same effect in the more advanced progress of the affection, when the granulations have become developed. The cicatrix resulting from its use is far more solid than one obtained from any other mode, and is not formed at the expense of the integuments of the edge of the wound, but from this new tissue. It cannot be used, however, empirically, for if the inflammation be very severe and phlegmonous, it may become aggravated by the chloride, which may delay the separation of the eschar. So, too, it cannot be used to arrest gangrene, arising from excessive phlegmonous inflammation. The strength of the solution employed must be regulated by the degree of irritation which it produces. This should be but slight—subsiding in ten minutes or a quarter of an hour. It acts far more efficaciously when the mucous substance of the skin is laid bare, than through the epidermis. A pledget, having several apertures made in it, and thickly spread with Galen's cerate, is applied over the whole surface of the burn. Upon this a mass of charpie, at least two inches in thickness is laid, and kept saturated with the chloride—the whole dressing being covered with a dry compress and bandage. The charpie must be moistened with the chloride six or eight times a day, and the whole dressing renewed every twenty-four hours. In winter, or when the prostration is great, the applications should be warmed; and, when the surface of the wound is extensive, portions of it should be dressed as fast as exposed.

The author was led to the use of the chloride of soda (which is better than that of lime) in burns, from having derived so much benefit from its employment in the treatment of ulcers. The rapidity of the cure is very great, and under its use superficial burns, even of a very great extent, are not found to be fatal. He thus sums up the benefits derivable from this application.

1. The pain is relieved, the inflammatory action and excitement of the nervous system diminished, and re-action prevented. 2. The cure is prompt. 3. The fall of the eschar is facilitated. 4. A better cicatrix results. 5. Many patients by this means may be relieved, who by the ordinary modes of treatment would have died.

Many useful observations upon the importance of position and bandaging, as preventives of the formation of vicious cicatrices, and upon the operations necessary for their removal, will be found in the work. The author has found considerable pressure very useful in cicatrices accompanied with tuberculous swellings, or enlargements of tissue, whether resulting from burns, or other solutions of continuity—as also in relieving the preternatural redness of cicatrices. The compression must be very considerable, (sometimes by means of leaden plates) and continued long after the apparent necessity for it has ceased.

### XII. OBSERVATIONS ON BLEEDING FROM THE ARM.

M. Lisfranc gives a detailed description of the usual course of the veins at the bend of the arm, and then alludes to their frequent irregularity.

"Everybody knows that the direction, number, and volume of these vessels are subjected to great variety; but it is a very remarkable and important fact, that the nearer the veins are placed to the external side of the limb, the seldomer do we find nervous filaments in their vicinity. The musculo-cutaneous nerve disengages itself from between the brachialis anticus and the biceps at about the middle of the tendon of the latter. Above this point I have never found a nervous filament around the median-cephalic vein. From the anatomy of the parts it results—1. That the upper part of the median-cephalic vein offers the most eligible point for venesection. 2. That in subjects, whose muscular system is well developed, the supinator radii longus covering, during the pronation of the forearm, the musculo-cutaneous nerve and the tendon of the biceps, we may bleed a little lower down. 3. In cases where the muscles are small, to the pronation of the limb, we must add slight semi-flexion. 4. When we cannot open the median-cephalic, we must select another vein, in the following order:—the continuation of the cephalic; the radial cutaneous; the median. But we must not omit to observe, that if this last vessel crosses the interspace formed by the supinator longus and pronator teres muscles, it will be always in contact with nervous filaments, whose lesion would be inevitable, and that, in subjects in whom the muscular system is slightly developed, the radial artery, then seated immediately beneath the ante-brachial aponeurosis, would be in danger of being wounded. The vein then should only be opened when it is seated externally or internally as regards this space. The great number of filaments of nerves accompanying the superficial ulnar veins seems to forbid our opening these; and where choice is only left between these and the median basilic vein, I prefer the latter. Whenever I have been lecturing upon the subject of bleeding, I have always requested any of my pupils, who may have had the median basilic vein opened, to shew me the cicatrix; and, astonishing to say, I have always found this situated at the point where the vein crosses the artery—the vessel being here rather more projecting than elsewhere. It should be opened on the outer side of the artery: but when its anastomosis with the median cephalic takes place too near the inner condyle to admit of this, we are obliged to open it on the inner side, though at the risk of puncturing the median nerve." 264.

Surgeons would seldomer renounce bleeding as impracticable because the vessels were not discernible, if the ligature were allowed to remain applied to the arm for a longer period than is usual—say half or a whole hour, during which time the patient should frequently contract the muscles of the arm. Placing the limb in a warm bath may sometimes succeed, but, at others, it has the effect of reddening the skin, tumefying the cellular tissue, and thus rendering the vessels still more obscure. The subcutaneous veins, especially those of the lower extremity, are charged with much less blood in the morning, after the patient has passed the night in bed; and, before bleeding can be practised, it may be necessary to exercise the parts somewhat.

Anatomical examination proves that the volume of the vascular system is proportionate always to that of the muscular; and, thus, women who live in idleness, as regards the use of their arms, will be found to have their veins always flattened, covered with an abundance of cellular tissue, and very difficult to open. It is in these cases, in opening the median basilic, we risk wounding the artery, to which the vein seems, as it were, almost attached.

When veins cannot be elsewhere found, and bleeding is yet required, we must remember that the cephalic is constantly to be found in the interspace of the deltoid and pectoral muscles, where an incision may be practised, and the vein then opened.

As often as possible, the vein should be opened *below* the cicatrices of former bleedings, as pathological anatomy proves that these sometimes contract, or even obliterate the vessels. When a vein cannot conveniently be opened elsewhere, we must not hesitate to open an old cicatrix. When, however, a second venesection is required, shortly after a former one, it is better to make a new opening in a vein, as manipulating the old one too much, in order to produce a renewal of the bleeding, may give rise to a phlebitis.

### XIII. On the Tying of Arteries.

For the compression of arteries during operations, M. Lisfranc prefers the fingers to the tourniquet.

"The mode of compression, beyond dispute the best, is the application of the fingers, unaided by any instrument, upon the track of the artery. When the vessel is superficial one hand suffices; but two are essential when it is deep-seated. The fingers, employed alone, presenting as they then do mutually opposing points, are the best means that can be employed for the suppression of hæmorrhage from the arteries traversing the substance of the lips, the ears, the alæ of the nose, &c. So, too, in the extirpation of a tumor having a delicate cyst, and adhering firmly to the skin, which has much diminished in thickness, if hæmorrhage occurs from the division of a great number of small vessels, the progress of the operation will be obscured and interrupted, unless compression, by means of the fingers, be made around the incision. The instrument invented by Lusardi is, however, preferable in operations about the eyelids. This mode of compression is the sole applicable one, when we desire to compress the abdominal aorta, the iliacs, the carotids, &c. It is more certain than any other, because the assistant feels the vessel while he suspends the course of the blood through it. If the fingers are displaced, an instant serves to replace them in—

an advantage attendant upon no other means. So, too, accordingly as the ligature of the arteries may require a diminution or cessation of such pressure for a while, it can at once be re-established if necessary. The fingers, too, compress a much less space, and do not bruise the tissues concerned like the pad of a tourniquet does. It will be objected, perhaps, that I give the preference to a means which requires much practice to employ, and that few persons can be found able to compress with their fingers alone for a sufficiently long time. This is purely imaginary. Why do persons fail in the endeavour? Because they press with a hundred times more force than they need—for, it is certain, that a very slight degree of pressure is required to arrest the flow of blood through the largest artery, and thus the assistant cannot become fatigued." 278.

All the steps practised in the operation for securing an artery are amply detailed, but they need not other notice here. Some remarkable cases are also given, to one of which we may allude—*Ligature of the External Iliac for an Aneurysm of the Femoral Artery*. The aneurysm was situated where it gives off the profunda. With great pleasure we quote the following admirable remarks of this celebrated surgeon respecting operations.

" In spite of all my certainty of accomplishing it, which my habit of performing operations, and demonstrating them to others, during eighteen years, has given me, I did not think it right to practise so important a one as this, without examining anew parts which I had so often lectured upon. By acting thus you take with you into the practice of surgery clear and precise ideas, and will not resemble those rash operators who impudently undertake anything, without considering what they are about to do. I entreat you never to act otherwise, when serious operations present themselves to you, the bad execution of which may risk the safety of the patient who has entrusted himself to your care—and then, even if a misfortune does occur, you, at least, have not to suffer from the consciousness of having neglected those precautions, which humanity demands at your hands. More than most others, I might, perhaps, have dispensed with such a precaution—yet, without any hesitation, I dismissed all vanity on this score, in favour of the interest of my patient." 301.

He thus describes his mode of exposing the artery.

" The external extremity of the incision was situated at two lines above, and an inch on the inner side of the anterior superior spine of the ilium, and the internal extremity at an inch and one-third on the outer side of the spine of the pubis, and about an inch above its level. I thus avoid, as far as possible, the inconvenience attached to Abernethy's operation—the perpendicular section of the abdominal muscles, encreasing the subsequent liability to hernia. I also avoid the too great length of the incision recommended by Cooper, which exposes the spermatic cord, the epigastric, and circumflexa ilii arteries to risk of injury. When I had completed the incision of the skin, fascia superficialis, and aponeurosis of the external oblique, I laid aside the cutting instrument, in order that I might draw aside the muscular fibres with my fingers—dividing some fleshy bundles with a probe-pointed bistoury. I detached the fascia transversalis below, and then opened it. Next, employing the palmar surface (instead of the points) of my three middle fingers, I pushed back the peritoneum in order to expose the vessels. I did not employ a grooved director in detaching the slight aponeurotic cellular expansions which serve as a sheath to the vessels, but employed the nail of my index finger, as more convenient and less likely to injure the peritoneum. I could now easily feel the artery, and distinguish its relations to the vein and nerve, and, by stretching the edges of the wound, I was enabled to see these quite distinctly." 303.

The peritoneum presents far more resistance than, from its thin texture, one would be led to expect, and, in raising it with the flat surfaces of the fingers carefully, no danger of its rupture exists. The case exhibited forcibly the greater difficulty that exists in operating upon the living subject, than in experimenting upon the dead. The man was a muscular subject, and the contractions of his muscles narrowed much the margins of the aponeuroses, and impeded the movements of the fingers, when raising the peritoneum. The struggles of the patient, also, thrust the viscera, fatty tissue, and the peritoneum, into the edges of the opening, and thus added much to the difficulty. The pulsation of the artery, on the other hand, is a great guide and assistance. The operation, including the intervals of delay caused by the struggles of the patient, and the examination of the cessation of pulsation in the tumor, only occupied a quarter of an hour.

The patient was bled several times during the first few days after the operation—cerebral and pulmonary congestion having become manifest. The ligature came away on the 21st day. The wound continued long unhealed, and, even at the 92nd day, a fistulous opening remained. Eventually this healed up, and the man perfectly recovered.

Eighteen months after the operation, no pulsation could be felt in the femoral, popliteal, posterior tibial, or dorsal arteries.

### XIV. False Primary Aneurysm.

M. Lisfranc once met with a case of fracture of the leg, in which the tibial artery was wounded, and an abundant infiltration of arterial blood into the substance of the limb occurred. This was accompanied by distinct pulsations in the course of the artery, and at the seat of fracture. Authors who state that diffused aneurysm is always unaccompanied with pulsation, are therefore in error.

Sometimes, after the wounding of even large vessels, hæmorrhage does not result for several days, the immediate effusion of blood being probably prevented in these cases by the application of a muscle over the small arterial opening. In other cases, a musket-ball has irritated the walls of an artery, and these have only given way as a consequence of subsequent inflammation. When great tumefaction of a limb occurs some days after an accident only, it must not be indiscriminately attributed to inflammatory action, as it may have resulted from the separation of an eschar from the walls of the injured vessel. The rapidity with which the swelling occurs is a better guide than the mere epoch.

When a ball has penetrated deeply into the tissues of the arm (*e. g.*) and hemorrhage subsequently occurs, the arteries, however, still pulsating below the wound, ought we to tie the brachial artery above the seat of injury? After such an operation the author has often known the hæmorrhage reproduced. If the wound is not too recent, if granulations are present, and if the inflammatory process is still going on, let the orifice of the wound be plugged up; and, as in such a state of the parts, the blood will probably be unable to infiltrate, it may accumulate and coagulate in the track of the wound, and become a means of compressing the bleeding artery. In about a week we may gradually evacuate the coagula. This means has been repeatedly employed with uniform success by M. Lisfranc.

### XV. NECROSIS AND CARIES.

*Necrosis.*—The author impresses upon his readers the importance of giving every possible chance to a limb, prior to determining upon an operation. He also cites, with great approbation, the researches of M. Malespine upon scrophulous necrosis. The spongy tissue of the bones is infinitely more frequently affected in this disease, than the compact. It almost always commences within the substance of the bone, rarely at its periphery, and still more rarely at the soft parts. It extends from the centre towards the circumference, and is arrested at the periosteum and cartilages. These parts form a "reproductive envelope," contained within which is found a sequestrum of greater or less size.

"This envelope is thus formed. The periosteum thickened and tumified, by reason of its function of secondary ossification, sends a fibro-vascular prolongation beneath the articular cartilages. This prolongation, which in the normal condition is not visible, becomes so under the influence of the phlegmasia induced by the presence of the sequestrum. It is adherent to, or rather intimately blended with the cartilaginous portion, which it lines. Sometimes it is red and fragile, at others considerably indurated. However this may be, it always accompanies all the phases of the reparative process, so that the primary cartilage covers the secondary ossification. It results from this curious disposition of parts, that these cartilages, placed at the boundary of the disease, are continuous with the periosteum, of which they may be considered an appendage—that after the cure, whether spontaneous or produced by the extraction of the sequestrum, they are always found at the periphery of the regenerated part—that they thus constitute at once a bond of union between the new production and the neighbouring bones, and smooth surfaces by means of which the various motions may be executed." 340.

The investigations of M. Malespine have also convinced him that the cavities, often found in the spongy texture of the bones of children, are not encysted tuberculous disease of the bones, as they have been represented to be—but the last stage of central necrosis, in which the sequestrum has become wholly or in part absorbed.

*Caries.*—Many practitioners feel surprised at the great success they meet with, in some cases, in the employment of the actual cautery, (which is preferable to the potential) and their complete failure in others, apparently identical. This arises from using the means empirically. It is improper to employ it when the soft parts are in a state of acute inflammation, for the bone participates in this, and it should be previously allayed. When the remainder of the bone is found, by examination with a probe, to be very soft, antiphlogistics are required, if the patient is strong and inflammation present. Otherwise, bitters, iodine, &c. may be useful; the cautery would prove hurtful where this softened state is extensive and considerable.

### XVI. Nervous Ophthalmia.

The chief symptoms are photophobia and lachrymation. The conjunctiva is somewhat reddened, but there is no change in the cornea or inner coats of the eye. The antiphlogistic treatment fails in these cases. The author has often found the smearing a little moistened, good *extract of belladonna* upon the temples, and around the base of the orbit night and morning, has effected, in a few days, the cure of cases, which had long resisted other means most obstinately.

### XVII. Indurated Tumors situated within the Substance of the Eyelids.

These occur especially in women, reaching sometimes a larger size than that of a bean. They are hard in texture, and placed near the margin of the eyelid. The skin covering them may remain healthy, or become inflamed, or even ulcerated, while the corresponding portion of the mucous membrane is found also of a deep red, or ulcerated. The accompanying inflammation must be met with antiphlogistics, but these will not usually dissipate the tumor. To effect this, after the inflammation has subsided, or if it has never appeared, the iodide of lead, (a better application than the hydriodate of potass,) should be applied. If suppuration occurs, a very minute aperture should be made. When the inflammation is trifling, a very slight application of a point of nitrate of silver, every six or eight days, to the reddened or ulcerated part, will be found useful. One application is usually sufficient, but three or four may be required. Extirpation must be had recourse to, where the affection obstinately refuses to yield to these means.

### XVIII. General Rules for the Extirpation and Removal of Tumors.

The following general principles, M. Lisfranc reprints from his work on Operative Medicine.

1. "A rule never to be lost sight of, is, that the cutting instruments should be used with a saw-like, rather than a pressing movement. I have found this one of the most difficult principles to impress upon the pupils, in my courses of operative surgery.

2. "The integuments should be stretched by the palmar aspect of the hand in an inverse direction to that in which the incision is to be made. When the operator wishes to make his incision from left to right, he should stretch the integuments by means of his left thumb towards himself, and by the ulnar border of the hand and the index and middle finger in the opposite direction. A more complete tension and a more ready and clear incision of the parts can then be made.

3. "To expose a superficial cyst, or one having very thin walls, or to open a hernial sac, the operator should stretch the tissues with the palmar aspect of his thumb and index finger, carrying his incision between these, nearly parallel to their axes.

4. " When the skin is very moveable the surgeon requires an assistant to produce the requisite tension.

5. " When by stretching the skin, parts which serve as a guide for making the incisions would be displaced, (as the raphe of the perineum, and some of the folds of the skin at the articulations,) the integuments should merely be steadied in their situation by the three middle fingers, pressed perpendicularly upon them.

6. " The incisions should be made parallel to the axes of the muscular fibres. If, however, a lesion of the large vessels or nerves is feared, we must cut in the direction of the axes of these latter. In operations about the forehead the incision should be carried transversely, in order that the wrinkles of this part may conceal the cicatrix. The providing a free exit for the pus, and the prevention of the interference of the cicatrices with the movements of the parts, must also guide our operations.

7. " The incisions should be commenced and not terminated nearest the origins of the nerves of the part. By interrupting the communication with the sensorium at once, any subsequent section of the nerves, that may be necessary, will be attended with much less suffering, than if the communication still existed.

8. " If dangerous lesions are not feared, the incisions should be continued a line or two beyond the extent of the tumor, giving greater liberty to the surgeon, and preventing the necessity of afterwards enlarging the incision, in case the tumor extends farther than was expected, which is frequently the case.

9. " When it is feared that superficial parts may be injured, we must divide the tissues horizontally, by means of a bistoury cutting on its convex edge. In these cases slowness of procedure is of the first importance.

10. " When two incisions unite but by one of their extremities, the second should always terminate in the first. If it commences in it, the skin, already divided and moveable, is not sufficiently stretched to prevent its receding before the scalpel, which divides it imperfectly and with difficulty." 382-4.

The author gives ample directions for making the various description of incisions; and offers some very interesting observations upon the difficulties which may occur during the removal of tumors, and the means of surmounting them. Our want of space prevents our doing other than refer to these, as well worthy the attention of those engaged in the practice of operative surgery.

A case is related of the extirpation of an encysted tumor, the size of a fist, extending some inches downwards from the mastoid process—so as to be in juxta-position with the " three carotid arteries." M. Lisfranc commenced the operation, by what he calls a preliminary incision, carried through the whole longitudinal diameter of the tumor. This proceeding he recommends as much facilitating the future steps of the operation, and illustrates it by alluding to the much greater ease with which the peritoneum can be separated from the walls of the abdomen, when an incision has been made into this cavity, than when none such has been premised, The cyst was easily detached from the sterno-cleido, larynx, &c., and with more difficulty and great caution from the important vessels with which it was in contact. The cure was complete in fifteen days.

We conclude this chapter with the following extract.

" Is it possible to diminish the size of an encysted tumor containing fluid, so as to render its extirpation more simple? In proportion as this tumor has become developed, it has drawn towards it the surrounding skin, which thus becomes displaced in proportion to the degree of the projection of the tumor. Let the cyst be punctured at its least dependent part, so that it does not empty itself completely, and escape from under the scalpel during the dissection. At least half the fluid will flow out, and the tumor will become diminished in size. The integuments, now less on the stretch, will to a considerable extent recover the position they occupied when the cyst was but slightly developed. It is evident that the extent of skin to be cut through will be much less, the dissection less difficult, the pain less severe, and the wound less extensive. We may mention that we have derived great advantage from this plan in the puncture and injection of hydrocele." 412.

### XIX. Treatment of Lupus.

Caustics fail in this affection frequently because they are applied with so little discernment. Prior to their use inflammatory action must be subdued. When this has been relieved, the *proto-nitrate of mercury*, which rather modifies than destroys the surfaces it is applied to, changes the action of the sore, and secures its cicatrisation. But, whenever an excess of irritation is excited, this must be first allayed ere benefit is derivable from the application. To subdue this, general bleeding, not leeches, must be employed—for, the bites of the last, even when placed at a distance from the seat of the disease, will often degenerate into foul ulcers. Three or four ounces of blood, taken at as remote a point from the disease as possible, will act revulsively; but, if the irritation is great, depletory bleeding will be required. The caustic may be required to be applied every week, until the foul character of the sore is removed, and a mere simple ulcer remain, for which it would be misplaced and hurtful. When the ulcer is very large, the caustic need only be applied at certain points, and yet the remainder of the diseased surface will become modified, though untouched by it. The author alludes to a patient, who had two phagedænic sores, one on the thigh, and the other on the abdomen. The first only was touched with the proto-nitrate, and yet the other, which had been long stationary, underwent also an important modification and cicatrised.

Relapse in this disease is frequent, but much less so when judicious internal treatment, proper attention to diet, and an occasional bleeding after cicatrisation, are attended to. If it have long existed an issue should be opened at some distant point.

A general remark may be made, that dartrous and herpetic maladies are too generally treated by excitants, and many more would be cured if bleeding and emollients were employed as substitutes, or at all events as preparatives for these. So, in treating various forms of ulceration, the inflammatory complications are overlooked, and the most powerful means fail. Depletion much assists the effect of mercurials, united with sudorifics, in the treatment of obstinate lues. Many ulcers of a very suspicious character, when not surrounded by very extensive induration, are cured by the combined use of depletion and caustic. Ulcers of the tongue, which would otherwise have been condemned to an operation, have been frequently cured in this manner.

### XX. Sanguineous Congestion.

In this chapter M. Lisfranc seeks to make an application of the fact, that congestion is not inflammation, nor to be treated as such. Thus, wounds, ulcers, &c. as long as the inflammatory condition continues, proceed very well under antiphlogistics and emollients: but, when this condition is subdued, they become stationary if such be persisted in. Although this fact is generally well known to surgeons, yet, he says, interminable and debilitating suppurations, œdema, induration, hypertrophy, and difficult cicatrisation, are frequently seen to arise from neglecting it. Compression, and a mildly stimulating mode of dressing are called for. But why should not the same principles be applied to the treatment of visceral inflammations, which may, in the same way, be ill-treated by a persistence in the debilitating regimen and sanguineous evacuations?—especially old gastro-enteritis.

### XXI. OBSERVATIONS ON HERNIA.

In reference to the statistics of the mortality from hernia, M. Malgaigne found that, out of 183 operations performed in the Parisian hospitals, between 1836 and 1841, there were 114 deaths.

The author is a strong advocate for the *taxis*, having usually found it succeed, when confided to skilful hands. If four days have elapsed it should not be used, even though there be no local or general symptoms of gangrene—for these are not always present even when it exists. When the pedicle of the hernia is thin—the neck of the sac very narrow—the tumor very hard—or the constricted parts are the seat of inflammation—the taxis is dangerous. During the employment of the taxis, the walls of the abdomen are usually recommended to be placed in a state of relaxation. M. Lisfranc objects to this for the following reasons.

"When the parietes of the abdomen are relaxed, they are applied upon the viscera contained in its cavity. The capacity of the latter is diminished, and the displaced parts are returned with less ease. 2. The relaxed walls of the abdomen will yield to the fingers during the attempt at reduction. 3. The relaxation of the walls of the abdomen prevents the formation of a hernia, while a tense condition tends to produce it.

"I place the parietes in a state of moderate tension, which facilitates the reduction. A comparison may illustrate what I mean. Endeavour to pass the clenched hand through an opening in a piece of cloth, &c. which is not kept on the stretch. It yields before you, and you effect your object with difficulty. If, however, it be stretched in the slightest possible degree, the opening will be passed with the greatest ease. But it will be objected to me, that this tension will narrow the abdominal rings. I reply—1. That it is only a moderate degree of it that I recommend. 2. That the objection does not apply to the crural canal. 3. That the muscular fibres around the inguinal canal are not placed so advantageously for the production of this constriction as may be supposed. 4. If in a thin subject, after the inguinal hernia is reduced, and the canal is spacious, the tissues covering its orifice are pushed through it by the finger, it will be found that the finger is not more compressed during tension than during relaxation of the abdominal parietes." 442.

As to the length of time during which the taxis should be applied, M. Lisfranc has frequently continued it for an hour, and has never seen ill-consequences result from so doing.

In effecting the reduction, M. Lisfranc places himself on the outside of the patient, and above, instead of below the tumor—drawing, as it were, the hernia towards him, and having in this position greater facility in directing it along the axis of the canal. In difficult cases he is obliged to try the ordinary mode as well.

Among the most useful means of facilitating reduction, the author considers the application of leeches to be one. They should not, however, be applied to the hernial tumor itself, as they render the skin too sensitive and the taxis more painful; and, owing to the oozing of blood from the bites, more difficult. Ecchymoses are also formed, which impede and obscure the subsequent steps of the operation. They should be applied around the base, not upon the tumor. He does not use the *tobacco* enema, nor does he consider the application of *ice* to be at all relied upon—while it sometimes produces gangrene of the skin.

*Purgatives*, after the reduction or operation, if stercoral collections exist, may be required, but, if there is any tendency to inflammation, they must be rejected, until this has been relieved—an effectual means of treating which, consists in the application of layers of mercurial ointment over the entire walls of the abdomen, as recommended by M. Serres d'Uzès.

### XXII. On Fistulous Sores and accompanying Indurations.

As fistulæ may be the cause of the indurated state of the parts which surround them—so frequently indurations maintain the open state of fistulæ. Thus, M. Lisfranc relates cases of induration and callosity of parts accompanied by fistulæ, in which the latter were relieved in proportion as the former were removed by antiphlogistics, emollients, compression, iodine, &c. When, however, the fistula still continues unhealed, an injection of the *chloride of soda* seems to have great power in inducing a plastic secretion, and causing cicatrization of such parts of the skin as have become denuded of cellular tissue. If the injections are used for several days without beneficial effect, they should be suspended, and compression then often proves of advantage. They usually fail because they are not used perseveringly enough, and the alternations with compression have not been frequent enough—say, six, eight or even ten times repeated. But the great error in the treatment of all chronic affections is this want of perseverance in the use of remedies, and the hasty adoption of a variety of new ones, before any one of the number has had a sufficient trial. If the fistula be a recent one, it may be cured by mere emollient injections, and these will sometimes succeed, even in obstinate cases, when more active measures have failed. The solutions of nitrate of silver, or of the protonitrate of mercury, are also valuable injections. This last must be cautiously and sparingly used—for the mere touching the fistula with it in a portion of its track, will, in many cases, induce the uniting process throughout its course.

### XXIII. The Resolution of Indurated Tissues by Incisions and Scarifications.

It was formerly believed that the indurated tissues, seated around fistulæ, old ulcers, caries, &c. would not, if saved in amputation, be ever capable of restoration to a normal condition. Many also believed they might degenerate, and cause long-continued suppurations, &c. These opinions gave rise to the practice, which M. Lisfranc also long followed himself, of including these indurated parts within the sphere of the operation—and thus, *e. g.* if the leg were so affected to a considerable extent, the amputation not of the leg, but of the thigh, would be determined upon. An observation made by Ambrose Paré, and his own practice in white-swelling, led him to believe that the loss of blood, and the subsequent production of inflammatory action in these tissues by means of incisions, might prove the means of restoring them. By trying experiments first on a small, and afterwards on a large scale, he soon found this to be the case. The diminution in the size of the parts, which will subsequently thus be produced, must always be borne in mind, in apportioning the quantity of skin to be left as a covering to the stump. If a violent inflammation seize the wound, these tissues, endued with but a feeble vitality, may fall into a state of gangrene. To prevent this, care must be taken to prevent the attendant inflammation reaching too high a pitch, by the use of leeches, cataplasms, and, if suppuration have not commenced, by revulsive bleeding. In a week, and in some cases in a fortnight, the indurated tissue often reacquires its normal condition. The incisions may sometimes be required to be both long and deep.

*Scarification* of the indurations surrounding ulcers had not been practised since Paré's time, until revived by M. Lisfranc. They are not to be employed indiscriminately, or practised too close to each other. Leeches and poultices may be required to subdue any excess of inflammation they may be followed by; but, there are other cases, in which the excitement induced proves insufficient to produce the resolution of the induration. They must not be repeated until the inflammation produced by the former ones has subsided, and the case does not seem to be progressing. If the tissues have become somewhat softened, but still continue enlarged, compression by means of a bandage and agaric is required—to be again replaced by scarification if requisite.

The author considers that the therapeutical employment of inflammation in the practice of surgery, (*i. e.* the excitement of an acute for the purpose of subduing a chronic inflammation), insisted upon by Dupuytren, is susceptible of a far greater application than it has yet received.

### XXIV. Of the Simple or Atonic Ulcer.

These ulcers are termed *atonic* from the supposed debility of the tissues in which they occur; and in support of this opinion of their nature, it is remarked that they are seated especially on the lower extremities—the point the farthest removed from the centre of circulation, and that they occur seven times out of ten upon the left rather than the right—the former being the weakest member. The author rejects these explanations; for, if the distance from the centre of circulation is to explain the production of the ulcers, we ought to find them more frequently seated upon the feet and toes than upon the legs. During his very extensive experience, he has observed, that many of the patients who had ulcers on the left leg were also left-handed, and, when this was the case, the left side of the body had frequently acquired a much greater strength and development than the right.

"The simple or atonic ulcer, is, according to my views, a gangrenous inflammation, *sui generis,* produced by the slowness and impeded state of the venous circulation. In proof of this: 1. Every one admits that the varicose ulcer is produced and maintained by a stasis of the venous blood. Its stagnation in the dilated vessels produces an inflammation, for the most part slight, which is followed by gangrenous ulceration. Acting more feebly, upon the venous system in an undilated state, the same cause produces the simple ulcer. When recent cicatrices give way, they usually become blue or even black, and it is evident that their rupture arises from stasis of the blood. 2. Supposing the crural vein (*e. g.*) to be wounded at the superior part of its course; you apply compression to restrain the hemorrhage, and thus arrest the passage of blood through the vessel. The stasis of the venous blood increases the volume of the limb, it becomes painful, hot, and then gangrenous—although the crural artery performs all its functions at liberty. M. Gensoul has tied the crural artery in cases which otherwise would have required amputation, and thus, by diminishing the quantity of blood which enters the limb, has prevented the stasis in its venous circulation. 3. In the treatment of ulcers every means for facilitating the venous circulation is employed—such as position, bandages, strapping. 4. Why are ulcers found so much more frequently upon the lower extremities? Why do we find them so much more frequently upon the left leg, and especially between the calf and internal malleolus? Because the venous circulation is performed with greater difficulty in the lower than in the upper extremities. Because the sigmoid flexure of the colon, often filled with fæcal matters, and crossing the left external iliac vein, impedes the circulation in it in a remarkable degree, upon the left side. Moreover, the two primary iliac arteries, by crossing the left iliac vein, produce the same effect of embarrassing the circulation in this latter vessel, not only by reason of the pressure they exert upon it, but by the impulse conferred upon the fluid they contain. M. Serres first demonstrated that the internal saphena vein is destitute of valves between the internal malleolus and the lower portion of the calf, and, indeed, in many subjects there are no valves, and in others only two between the ankle and the knee." 507-9.

The success which has attended the section of the saphena vein in bad ulcers, which would otherwise have required amputation—attacking any phlebitis that may arise by leeches, &c.—is another proof of the truth of the views adduced by the author.

That the inflammation producing the ulcer is a gangrenous inflammation, he considers proved by the fact that, under the phlyctenæ, usually preceding the ulcer, a brown gangrenous spot is found—that the progress and attendant odour are those of gangrenous sores—that eschars are formed if the progress of the case be rapid; and, when this is not the case, by taking the ichorous pus between the finger and thumb, and moving it to and fro, a solid detritus, formed of very small portions of gangrened tissues, may be felt, and will be found to exhale the peculiar odour of these—that suspension of circulation of the blood in the crural vein will induce gangrene.

The author, speaking of the hæmorrhage resulting from varicose ulcers, says :—

"Violent hæmorrhages are found sometimes to complicate varicose ulcers, under which patients have sometimes sunk. In these cases, I have found at the necropsy, that the internal saphena was opened; and, upon dissection, it was seen to be thickened, and its internal coat inflamed. When its walls were cut across their circumference, in all their thickness, they did not collapse, and the canal, besides being anormally increased in size, remained patent—the section resembling that of an artery. Thus, it would seem very difficult for the vein to act upon the fluid which it contained, and, although provided with collaterals extending even to a great distance, it resembled, in fact, an inert tube, from which the blood, obeying the unresisted impulse of gravity, escaped without any obstacle, when the ulceration had taken place." 515.

Of the resorption of pus he says :

"The resorption of pus, which is too frequent from the surface of wounds, is very rare in ulcers. Experience has fully proved the truth of this. It is well known, also, that muriate of morphia, applied to a recently-blistered surface, is rapidly absorbed, but, if the exutory be of old date, then the substance scarcely, if at all, obtains admission into the circulatory passages. These contradictory phenomena have appeared inexplicable; but in old solutions of continuity, there exists upon the denuded surface a newly-organized tissue, which is not yet endued with the same degree of vital energy that it will eventually acquire—offering thus a frequently insurmountable barrier to external agents endeavouring to penetrate into the animal economy." 516.

As to the question of whether ulcers (old) should be indiscriminately healed up—the healthy condition of the principal viscera—the patient's general state of health, and the nature of the constitution in each case, must guide our practice. When we have determined in the affirmative, we must not accomplish it suddenly. An issue in the leg or arm should also be opened, and bitters, or mild purgatives, as the condition of the patient may require, should be given. If, in spite of all precautions, congestion or inflammation of some important organ does supervene, the cicatrix must be irritated by means of a stimulating ointment, a blister, or even boiling water.

*Treatment of Atonic Ulcer.*—Absolute repose, and a well chosen position of the part are essential. If the patient be not too feeble, and inflammatory action accompany the ulcer, a bleeding from the arm may be premised, and, even when mere œdema exists, attended with very slight inflammation, the same practice will induce its absorption. As further means of subduing inflammatory action, cleaning the surface of the ulcer, and removing the induration of its edges, emollient cataplasms are required: and Lisfranc has cured many atonic ulcers with these alone, and always continues them as long as cicatrization seems progressing. He has often found strapping the ulcers fail, though, in others, the practice has been attended with excellent effects. He deprecates the modern practice of abandoning the use of unguents, which are often so highly useful. Monesia, used in powder, or as an ointment, is an excellent stimulant application, and another, besides calomel, is a powder formed of five parts of starch and one of sulphate of alum—with which the surface of the ulcer is to be covered.

The surrounding *indurations*—which both impede the formation of cicatrices, and render them unstable—are to be treated, if inflammatory action exists in them, by leeches, applied at the upper part of the leg; and when this state has subsided, iodide of lead ointment, leeches in less numbers, and compression; and where all these fail, slight applications of the proto-nitrate of mercury to the ulcer itself, or scarifications of the indurations, are the means of relief. When an induration is limited in extent, but horny in texture, it is to be removed like an ordinary tumor.

Since 1825, M. Lisfranc has been in the habit of using the *chloride of soda* to expedite cicatrization. But for this to be useful, the sore must be clean, the granulations in a normal state of development, and cicatrization commenced. It is used on the same principles as in burns, and, when properly employed, results are often obtained in a week or a fortnight, which could not otherwise have been procured in five or eight weeks.

It is not less important to secure the solidity of a cicatrix than to obtain one, and the ordinary use of the laced-stocking is an impediment to this. It exerts an equable and considerable pressure over the whole leg. Upon taking exercise, an encreased volume of the limb ensuing upon muscular contraction, especially at the calf, the superficial venous circulation becomes impeded, and a stasis of blood produced. The stocking should then be less tightened at the calf than below it, and its descent may be prevented by attaching it to the drawers. The portion of a bandage applied to the calf might be composed of caoutchouc. In spite of every care the cicatrix too often gives way, and a more obstinate sore than ever is produced. These consequences would be far less frequent if the patient could repose the leg for a few months after cicatrization.

The attempt to obliterate the superficial veins supplying varicose ulcers has frequently given rise to most serious consequences. But this has been much less commonly the case since the consequent phlebitis has been treated with more skill and success. M. Lisfranc has found most excellent results attend the application of a great number of leeches immediately above the inflamed portion of vein, *i. e.* between it and the heart. The obliteration, however, should not be attempted except in most obstinate cases, defying all other means, and necessitating either complete inaction or loss of limb. All inflammation about the varices must be first removed, and six or eight weeks may be so employed. The usual site of the operation is just below the condyle of the tibia, or, if the vein be affected higher up, the lower and inner portion of the thigh is chosen—avoiding operating too near the abdomen, lest any attendant phlebitis may spread to within its cavity. We must act above the collateral veins, or a sufficient obliteration will not result to ensure the cure of the varices and ulcers. We must, too, avoid operating upon the dilated portion of the vessel, or too near the varicosed part, as a condition of chronic inflammation is often present, upon which we might induce an attack of acute.

M. Lisfranc has employed Guerin's subcutaneous section of the vein but once, and then with success—and he anticipates that this will prove an operation of great utility.

The operation M. Lisfranc himself performs consists in exposing the vein to about the extent of two inches, dividing it by means of curved scissors at each angle of the incision, and removing the portion so separated—an assistant by compression preventing the access of air into the vein, during its division. Light, graduated compresses are applied, and the wound is healed by the first intention. The patient is to be treated afterwards upon the same antiphlogistic plan as after a capital operation.

Even in simple atonic ulcer, unaccompanied by a varicose state of the veins—when the sore is very extensive—when it is impossible to produce its cicatrization, or that this is continually giving way—when the patient's life is in danger—or that he is compelled to inactivity during most of the year—obliteration of the vein, in the place of amputation of the limb, will be often attended with the happiest success.

Relapse of an ulcer after the section of the vein is excessively rare.

When patients, who had been formerly operated upon for varicose ulcers, have returned to the hospital, in consequence of other complaints, and have died from these latter—the autopsy has shewn the internal saphena obliterated from the point where the incision was made to the great toe—resembling the umbilical vein in the adult. Many of the collateral veins and their branches partook of the same condition. No trace of varices could be found upon the extremity, and the circulation was chiefly carried on through the deep-seated portion of the venous system. In a few patients slight dilatations have been observed near the obliterated vein ; but these have been prevented from increasing by the laced stocking, and have never caused a rupture of the cicatrix.

### XXV. THE WHITE-SWELLING OF THE JOINTS.

We need not occupy our space in detailing our author's account of the pathological anatomy of these joints, as our readers are familiar with the more masterly delineations of Brodie. But there are many other points of great interest in the present Chapter—and to these we proceed.

White-swellings may be distinguished into those in which a sub-inflammation is present, and others, in which no trace of this exists. In some cases the inflammation is latent, and in others acute—all which facts are very important, in a therapeutical point of view.

The author considers that they are in error, who state that the mere *engorgement* of the soft parts can always be easily distinguished from an enlarged condition of the bone. He long believed erroneously with others, that a firm or flinty consistence of the swelling was necessarily a proof of an affection of the osseous system. In such a case he now hesitates before he ventures an opinion—especially if the tumor do not present an irregular surface. By suitable treatment the induration often becomes moveable upon the bony parts—these last remaining nearly or quite healthy. In such cases, the disease has proceeded from without inwards—the deeper-seated soft parts being those last affected; and, hence, yielding to the means employed, they have become soonest cured, and the more external tissues, remaining indurated, are moveable upon them.

"There is one peculiar form of white-swelling, which, I believe, I was the first to describe. The joint is but little encreased in size, and the swelling gives, when touched, the sensation of an elastic or spongy body, such as the spleen or placenta, or a lipoma about to undergo degeneration. Here and there are formed small but very numerous abscesses, separated from each other. When these burst there is discharged, with the pus, a flocculent matter, produced by the death of the tissues involved. I have as yet never been able to cure any of these cases. At the autopsies I have found a lamellated tissue, traversed by a great number of vessels, and containing white granules, resembling tubercles. This tissue was absent at the points wherein the little abscesses had cicatrized, being replaced by ordinary inodular substance." 574.

He enquires whether it would, in these cases, be prudent to employ means to encrease the inflammatory action, and thus expedite the suppurative process. The peculiar tissue in question is at first superficially placed, and might it not be removed by operation, when it occupies but a limited space?

In cases of white-swelling it is very important to be assured of the condition of the thoracic and abdominal viscera; for, where diseases of any of these exist, they have sometimes been found to make progress in proportion as the affection of the joint becomes amended—even requiring that irritation should be reproduced in the latter. When the visceral affection is incurable, but stationary, we do not treat the white-swelling actively, unless it become dangerous; and, in fact, we may have to pay our attention especially to the external or internal malady, as either may become alarming, and, by this temporizing conduct, may often succeed in prolonging the days of the patient, when they would be cut short by a more vigorous procedure. Unfortunately the thoracic and abdominal affections are often *latent*, and resist our means of investigation,—so that, death may speedily follow the cure of the disease of the joint, in consequence of some unsuspected organic affection. Whenever the disease of the joint, without any obvious cause, undergoes many vicissitudes, so that, from being nearly cured, it relapses into as bad a state as ever, we must suspect something wrong in the system, although no special symptom be present.

Abscesses, formed *around* the white swelling, should be opened as promptly as possible, while, when formed *within* the substance of the engorgement, exit must be given to the pus as late as possible—providing the inflammation be not acute—that there be no danger of the pus penetrating the joint—and, that the skin be not denuded of cellular tissue. It is very easy to be deceived as to the existence of pus—while the sojourn of this fluid in contact with the indurations proves to be one of their most powerful solvents. The most experienced surgeons often have great difficulty in deciding whether a collection of fluid or pus be situated within, or external to the joint. The alternative of amputation decides the surgeon in determining to open the collection, and, if symptoms follow, which denote that the cavity of the joint is exposed also, he proceeds at once to amputate.

M. Lisfranc considers that, in general, much too little importance is attached to *regimen*, in *chronic surgical disease*. The ordinary, and sometimes an excessive diet is permitted, and the great aid in dissipating engorgements, derived from allowing patients, in some degree, to, what he calls, "live upon their own substance," (*i. e.* a starvation regimen) is lost sight of. He has repeatedly known these tumors, which have resisted every other means, to become dispersed after diminishing the diet by one third or a half. Exceptions will occur to this, when the patient is excessively feeble or scrofulous, and when the digestive organs suffer from the change.

If the patient be strong, and any acute inflammation exist about the joint, one or more bleedings from the arm, and afterwards leeches, will be required. But the periods of employing these must not succeed each other too rapidly—for chronic diseases by their duration frequently induce great debility; and inflammation, seated in these anormal tissues, is not susceptible of the same ready removal as when it occurs in healthy ones. Local emollient and anodyne baths, continued for two hours at a time, are useful. Although, by appropriate means, the inflammatory element may disappear in a month, it may persist in other cases for three, six, or nine months, and, to such cases, which may also have resisted mercury and barytes, we must oppose time, and the continued application of small relays of leeches. Eventually the moxa may be employed. It is rare indeed to find one of these swellings dissipated by antiphlogistics alone. A slight diminution of volume only results from the subsidence of inflammatory action.

After the employment of antiphlogistics, an interval must be allowed prior to commencing the use of stimulants—but the presence of occasional pain, or pain which has resisted depletion, must not deter us.

*Leeches* may be applied for other than the ordinary reasons. From three to six, or eight, placed around the base of the tumefaction, will cause a degree of heat and excitement in the swelling, when torpid, and favor absorption. Five different effects may result from their use.

"1. No effect may result. You wait three or four days and then re-apply them; and if this time you are not more successful, you abandon them—having thus ascertained that their action is too feeble to produce the degree of excitement sufficient to favor the resolution of the engorgement.

"2. The heat of the skin of the part may be slightly increased, and a slight pain felt. Avoid the application of emollient cataplasms, &c. for you will destroy the salutary excitation you have produced. Cover the swelling with a fine linen cloth, and you will soon observe its diminution—which will be often preceded by a superficial and scarcely perceptible ramollissement of its tissue.

"3. The size of the tumor becomes increased by two or three lines, a slight œdema existing in the subcutaneous cellular tissue. These circumstances astonish and frighten the patient, when he has not been prepared for them: but the surgeon congratulates himself upon them, for, shortly, not only does the tumor return to its primary size, but, in a day or two, it becomes diminished to the extent of several lines.

"4. The leeches may have produced a still greater excitement. The swelling has become enlarged, hot, and during a part of the day, painful, while the leech-bites become surrounded by a slight erysipelatous circle. These circumstances, too, augur well. Almost constantly, at about the end of forty-eight hours the erythema disappears, and a most marked diminution and amendment in the tumor supervenes.

"5. Sometimes ordinary erysipelas follows. We must at once attack it by applying a very great number of leeches, attention to regimen, &c.—in order that we may prevent the inflammation penetrating the subjacent tissues. When the erysipelas disappears, the strong excitation of the parts it occasioned, will be found to have expedited the cure in a notable degree." 593-4.

The leeches may be repeated, in these small numbers, whenever the amendment they produced becomes stationary. The bites must not bleed for more than a quarter of an hour, so that the patient does not become weakened by the loss of blood. They may be frequently renewed, but, as after a time, the economy becomes habituated to their use, they cease to produce the same effects. But if, in this case, we suspend their employment for a while, upon recurring to them, they are found to succeed as well as ever. They may also often be used with advantage in combination with, or in succession to other means, as compression, &c.

*Cupping*, also, may be substituted for leeches, and like them, acts as an antiphlogistic or stimulant, just in proportion to the quantity of blood drawn.

*Compression* is useful by preventing the too free access of blood to the tumor, and causing a slight degree of excitement at its surface. It is most advantageously applied so that it may extend an inch beyond the circumference of the tumor. If employed in improper cases, it may occasion inflammation or gangrene; and, in all, requires regulation in its various degrees, according to the stages and peculiarities of each. Thus, the mere application of diachylon, the use of bandages and agaric, and the application of leaden plates to the part, excite different degrees of compression. The compression may be re-adjusted every twenty-four hours, and its employment does not exclude the use of iodine ointments, and the various internal remedies. Even after the tumor has disappeared, especially when of a scirrhous consistence, compression must be continued for several weeks longer.

It is to be rejected—1. When considerable inflammation exists. 2. When the tumor, though small, is very hard, unequal, knotted, adherent to the skin, and especially if this latter be red or discoloured. When, however, the scirrhous mass has been removed, we may employ pressure. 3. When the tumor is in some parts indurated, and in others pultaceous.

The author believes Dr. O'Beirne's conclusions, concerning the utility of *salivation* in these cases, are too general, and too premature. This practice is attended with great success, only when inflammatory action is present, although it even then requires the aid of other means to effect a cure, as, after a certain degree of amendment, the case remains stationary. Mercurial inunction, carried to salivation, is equally useful as the calomel. When no inflammation of the part exists, the mercurial ointment, applied by topical friction, is an excellent solvent—but, when inflammation does exist, it must be applied in thick layers, to remain in contact with the part, as recommended by M. Serres, when it acts as an antiphlogistic, and not, as in the case of friction, as a stimulant to absorption. All friction with iodine ointments must be foregone as long as any inflammation exists. The ioduret of lead is the best preparation, but any that may be used requires watching, lest excitement to an injurious extent be produced. He has found iodine, and especially the hydriodate of potash, given internally, very useful—particularly in scrofulous cases.

*Blistering* must not be employed when even sub-inflammation is present, being as it is essentially stimulant. If blisters are placed *upon* the tumor itself, when the skin is indurated or adherent, acute inflammation of a most obstinate character will be produced, and which would have been avoided by placing them in the vicinity of the part. When the blister occasions an injurious degree of inflammation, we must meet this with leeches, &c. We must judge of the ultimate effects they are likely to produce by the degree of irritation that results.

The application of the *moxa* follows the same rules as the use of blistering; and its repetition has been found useful in some cases, wherein amputation was imminently threatened. When a repetition is likely to be required, the moxa must be of only a small size—somewhat less than a shilling.

The *seton* is the most exciting of all exutories, and must not be employed until the inflammatory action has subsided. It should not be passed through the substance of the engorgement, but on one side of it. It is only to be employed when all other excitants have failed.

There are cases of white-swelling, which after proceeding well on towards a cure, remain, at length, quite stationary, treat them how we will. In these we must abandon all active measures, contenting ourselves with hygienic precautions; and, sometimes in a few weeks, we may find the swelling much dissipated, and, even if still persistent, the various therapeutical agents will usually be resumed with much greater advantage, after such an interval.

M. Lisfranc states that, in his hands, the *muriate of barytes*, given in large doses, has been found very successful, the patient being also during its use confined to a vegetable diet, and water drink—upon which, however, he often gains both flesh and strength. He begins with six grains, and reaches, in some cases, as high as forty-eight grains per diem; while, at Marseilles, and in Italy, two drachms have been given in the same period. He alludes to many cases, in which the employment of this medicine has saved limbs, that must otherwise have been removed by amputation.

When the white-swelling dates from a *rheumatic* origin, it has been recommended to place an irritant, such as a blister, upon the joint itself. This practice is attended with the danger of fixing the locality of the inflammatory action; and M. Lisfranc prefers, in the case of the knee-joint, *e. g.* placing the blister at the upper and outer part of the thigh; and in following this practice he has met with great success. In some of these cases, the pain, which is so distressing, has disappeared under salivation, as if by enchantment.

After a cure the joint is not at once enabled to resume its functions. The patient has remained at rest during a long space of time, and pain attends his first movements. If this ceases in a quarter or half an hour, after attempting to walk, &c. we conclude it has been produced by mere desuetude; but supposing it is continuous, the patient must not yet abandon repose, and the various remedial means he has been employing.

Relapses were formerly very frequent, but M. Lisfranc has found them to be rare, since he has given his patients a padded knee-cap to wear.

This limits the movements of the joint, and supports it during their performance, prevents any stagnation of the fluids, and aids the resorption of any effusion that may occur. Atrophy of the joint sometimes follows, which may be persistent, or may disappear, as the general health improves.

Many cases are quoted to demonstrate amply the author's mode of treatment, concerning which he thus remarks :

"I am frequently asked by what plan I cure white-swellings : but these words offend my ear. I reply, that my method is dictated according to the various indications the cases present; and that under many circumstances, in order to obtain a cure, we are not only obliged to employ a great number of means simultaneously, but frequently to exhaust, as it were, all we possess. The disease may pass frequently from the acute to the chronic condition, and, in the majority of cases, the means which have at first produced great amendment, after a while, do not continue to do so, and require to be replaced by others. These are the principles which I shall again and again illustrate in the course of this work, for I feel the indispensable necessity of establishing them." 610.

### XXVII. Non-venereal Exostosis.

Scrofula, gout, rheumatism, scorbutus, and cancer, may give rise to this affection.

External violence is usually supposed to be a mere exciting cause of the development, and local fixation of some inherent virus ; but M. Lisfranc has so frequently seen contusions, &c. followed by the production of exostosis in persons in whom no such condition of the constitution existed, that he does not believe this view of their origin is a correct one.

Must we necessarily sacrifice a bone, because a tumor of suspicious character is situated upon it—provided such bone be not affected by necrosis or caries, or be not preternaturally softened ? Experience proves that, in the majority of cases, the encrease of its size is due to a mere encrease of its nutrition, occasioned by the irritative presence of the tumor—and that, after the removal of the latter, the exostosis usually remains stationary, or disappears more or less completely. Exostosis may or may not be accompanied by inflammation of the osseous tissue ; and the recollection that there are two opposite classes of cases, is highly necessary in a therapeutical point of view. The means to be employed resemble those proper for white-swelling ; but, in the present cases, the reproduction of the inflammatory action is infinitely more easy and more frequent.

Exostoses are often developed after apparently slight bruises, to which a requisite degree of rest and attention has been denied. Such injuries require the prompt and effective application of leeches, cataplasms, &c.

Several illustrative cases are cited.

We have left unnoticed two chapters : the one upon Furunculus, and the other upon General Rules for performing the various Disarticulations. The former contains nothing worthy of note, while the latter is too long for extract, and yet too condensed for abbreviation. With the exception of these portions, we have endeavoured to present our readers with a faithful transcript of M. Lisfranc's opinions. Many of these are original, many of questionable correctness, and all of importance, seeing the influence he exerts upon continental surgery—an influence, we are happy to say, likely, in the main, to lead to its great improvement. It is gratifying, also, to find one who has achieved so high a reputation as an operator, continually protesting against hastily having recourse to operations; and again and again declaring, that the highest province of surgery consists in avoiding rather than in performing them. "If," says he, "surgery is beautiful when she operates, she is infinitely more brilliant when she effects a cure without depriving the patient of any of his limbs—without plunging her scalpel into his quivering flesh—without causing the flow of his vital fluid." May this sentiment, avowed as it also is by so many of our own most celebrated surgeons, have its due effect in repressing that recurrence to rash and useless operations, which we fear is prevalent among some members of the profession even at the present time!

We are well pleased also to find our author strongly insisting upon another distinguishing feature of scientific surgery—its intimate union with medicine, indeed their indivisibility. But, truly, the multiplicity of operations can alone be prevented by such union, for the mere chirurgeon (we should hold such a one to his etymology*) never having become acquainted with the resources of nature and art, can never avail himself of them, and, unable to unravel the Gordian-knot, must content himself with severing it.

We cannot equally compliment our author upon his style, which is wordy, diffuse, and slovenly—so that all his precepts might have been compressed into a much smaller space, and expressed with much greater clearness. There is throughout the work, also, a continual querulousness. By his own account, M. Lisfranc is the most injured man living, for scarcely is there one of his various plans of treatment, but has been appropriated or misrepresented by others. Nothing short of a surgical autocracy we fear will satisfy him.

---

* χειρ manus, and εργον opus—literally, handicraft.

